# High-risk neuroblastoma in Mexico: from multimodal treatment to immunotherapy. Regarding the first case treated with naxitamab

**DOI:** 10.3389/fonc.2025.1520171

**Published:** 2025-06-18

**Authors:** Alberto Olaya Vargas, Haydee Salazar-Rosales, Marcela Caballero-Palacios, Nideshda Ramírez-Uribe, Gerardo López-Hernández, Erika Morales-Martinez, María Cervantes-Delgado, Liliana Velasco Hidalgo, Araceli Castellanos-Toledo, Rocío Cárdenas-Cardos, Ana Niembro-Zúñiga, Roberto Rivera-Luna, Alberto Olaya-Nieto, Jesús Ponce-Cruz, Jaime Shalkow-Klincovstein, Yadira Melchor-Vidal, Rodrigo Díaz-Machorro

**Affiliations:** ^1^ Department of Hematopoietic Transplantation and Cellular Therapy, National Institute of Pediatrics, Mexico City, Mexico; ^2^ Deparment of Oncology, National Institute of Pediatrics, Mexico City, Mexico; ^3^ Pediatric Hemato-Oncology Unit, ABC Medical Center, Mexico City, Mexico

**Keywords:** high-risk neuroblastoma, immunotherapy, naxitamab, Latin America, pediatric cancer

## Abstract

**Introduction:**

We report herein the case of a patient diagnosed with high-risk neuroblastoma (HR-NB), treated with naxitamab following suboptimal response to induction chemotherapy, becoming the first patient to receive this therapy at the National Institute of Pediatrics (INP) in Mexico. We discuss the clinical course, therapeutic approach, response to treatment, adverse events, and fatal outcome, focusing on the implications of immunotherapy access in low- and middle-income countries (LMIC).

**Case presentation:**

A 2-year-old male presented with a 3-month history of a left front-temporal mass and left-sided exophthalmos. Magnetic resonance imaging (MRI) revealed a poorly defined retroperitoneal and paravertebral mass, and a destructive cranial lesion involving the sphenoid wing and the orbit. PET-CT and MIBG scans confirmed widespread metastatic disease in bone, orbit, and intracranial structures, consistent with stage 4 neuroblastoma. Histopathology of a cranial biopsy confirmed poorly differentiated neuroblastoma. The patient was classified as high-risk based on age and metastatic disease, and underwent multimodal treatment including chemotherapy, partial tumor resection, radiotherapy, and anti-GD2 immunotherapy with naxitamab under protocol HITS-17-251. After five cycles, imaging showed complete response in cranial metastases. Adverse events during immunotherapy were mild (Grade I–II), including erythema, pruritus, urticaria, and transient hypotension, all managed with antihistamines and IV fluids. However, one month after the final cycle, the patient developed sudden neurological deterioration with cerebral edema and hydrocephalus. Despite intensive care, he progressed to brain death, with neurotoxicity suspected as the cause.

**Discussion:**

The primary objective of implementing novel immunotherapeutic strategies, in addition to improving event-free survival (EFS) and overall survival (OS) in patients diagnosed with high-risk neuroblastoma, is to reduce adverse effects and lower both short- and long-term mortality. It is worth noting that the current cost of anti-GD2 therapies available in Latin America is estimated to exceed $450,000 USD. In a country like Mexico, where the minimum daily wage is approximately $10 USD, access to these therapies remains unattainable for the majority of the population. Therefore, it is essential to highlight the importance and substantial clinical impact of immunotherapy on survival outcomes in patients with refractory neuroblastoma. This recognition should support advocacy for broader access to these therapies. Despite their high costs, the demonstrated benefits should outweigh the disadvantages, justifying efforts to make them accessible to all patient populations.

**Conclusions:**

Future studies in LMICs including Mexico and Latin America, must focus on optimizing dosing strategies to minimize adverse effects and improve both survival outcomes and the quality of life for patients receiving immunotherapy.

## Introduction

Neuroblastoma (NB) is the most common extracranial solid tumor in pediatric patients, originating from neural crest cells of the peripheral sympathetic nervous system (1). In Mexico, between 2007 and 2010, NB ranked tenth among malignant neoplasms in children and adolescents, with a prevalence of 1.9% and an annual incidence of 2.3 cases per million (2). Currently, no more recent epidemiological data are available for neuroblastoma cases in Mexico.

A key feature of NB is its ability to present with diverse clinical manifestations, ranging from localized disease to widespread metastases (3). The relevance of these clinical presentations lies in their influence on treatment strategies, which are risk-adapted and tailored to specific prognostic features that impact survival.

The risk stratification approach aims to minimize overtreatment in low-risk patients while intensifying therapy for high-risk individuals to improve survival outcomes. Two of the most critical features used in NB risk classification are patient age and MYCN gene amplification status, both of which are strongly associated with poorer survival rates (4). In clinical practice across Mexico, the diagnosis of NB in various centers is mainly based on the strategies recommended by the Children’s Oncology Group (COG) (**Table 1**). The most common primary sites of NB include the abdomen, neck, and thorax, with symptoms varying according to tumor location (5).

**Table 1:** Children’s Oncology Group (COG) recommended strategies for the diagnosis of neuroblastoma (NB).

Medical history and physical examination	
Most frequent symptoms	• Localized abdominal tumor: abdominal edema, pain, constipation, difficulty urinating. • Localized neck tumor: palpable lump in the neck, occasionally accompanied by ptosis, small pupils, and lack of sweating on the same side of the face as the lump. • Fatigue, indicative of bone marrow dissemination. • Others: fever, bone pain, bleeding and/or bruising.
Less common but possible symptoms	• High blood pressure, age-inappropriate elevated heart rate, persistent diarrhea.
Laboratory tests	
Complete blood count	• Thrombocytosis may indicate tumor growth in the bone marrow. • The results are also useful for determining the degree of kidney and liver function.
Urine sample analysis	• In up to 90% of NB cases, tumor cells increase hormone concentrations, which are broken down in the body into homovanillic acid (HVA) and vanillylmandelic acid (VMA), both of which are excreted in the urine. • HVA and VMA levels are abnormally elevated in NB patients and are used as indicators of insufficient treatment response or signs of recurrence.
Imaging*	• This group of tests includes: X-rays, ultrasound, computed tomography (CT), magnetic resonance imaging (MRI), and meta-[123I] iodobenzylguanidine (MIBG) scintigraphy
*MYCN* Oncogene	• Amplification of this gene by >10 copies is associated with unfavorable histology and prognosis.
Chromosome 11q deletion	• It shows an inverse correlation with the amplified MYCN gene; its presence is linked to an unfavorable prognosis.

** It is important to mention that the new updates regarding clinically relevant criteria for the diagnosis of patients with neuroblastoma include age, MYCN amplification, and ploidy; these are analyzed by fluorescence in situ hybridization and flow cytometry. Based on the emerging criteria, the analysis of loss of heterozygosity of 1p and 11q is also studied and analyzed by whole exome copy number.

## Prognostic factors used in Mexico for risk stratification

Over the years, more centers in Latin America have gained access to molecular diagnostic tools for NB (6); however, in Mexico, routine testing for MYCN expression has not yet been universally adopted. As a result, risk stratification in a significant number of neuroblastoma cases in the country is based primarily on imaging studies, age, staging, and histopathology.

The factors relevant to patient survival in NB include:

- Tumor stage and age at diagnosis: Prognosis is significantly more favorable for patients younger than one year with localized or minimally disseminated tumors (stages 1, 2, and 4S) compared to those older than one year with similar clinical presentations. Outcomes for children aged 1 to 2 years with disseminated disease (stages 3 and 4) are better than those observed in patients older than 2 years at the same stages (7).
- Histopathological data: Patients under 18 months of age, with tumors showing poor Schwannian stroma (poorly differentiated or differentiating, and low or intermediate INSS grade) have a favorable prognosis. Patients aged 1.5 to 5 years with differentiating stroma and low INSS grade also have a favorable prognosis. Unfavorable prognosis is observed in infants less than 18 months of age, with undifferentiated stroma and high INSS grade, those aged 1.5 to 5 years with undifferentiated or poorly differentiated stroma and intermediate or high INSS grade, and patients older than 5 years regardless of tumor type (8).
- Tumor cell ploidy: Hyperdiploidy (DNA index > 1.0) is associated with a favorable prognosis, whereas diploidy (DNA index: 1.0) correlates with poor prognosis, especially in infants under one year of age (4).
- MYCN gene amplification and chromosome 11q deletion: Both are associated with poor prognosis. MYCN oncogene amplification is defined as the presence of more than 10 copies (9).
- Neurotrophin and receptor expression: Three tropomyosin receptor kinase (Trk) receptors—TrkA, TrkB, and TrkC—have strong affinity for different neurotrophins essential for normal neuronal development. In NB patients, differential expression of these receptors influences the biological and clinical heterogeneity of the disease. TrkA expression is inversely correlated with disease stage and MYCN amplification, with high expression linked to favorable prognosis (10).

Based on these factors, up to 80% of children with NB in Mexico are classified as high-risk, and specific therapeutic approaches are implemented accordingly.

## Treatment strategies established in Mexico for neuroblastoma

For patients categorized as low-risk, observation alone is often recommended, as spontaneous regression is common (5). These patients are monitored through serial ultrasounds; if tumor growth exceeds 50%, surgical resection may be considered. Risk classification is further refined based on molecular biology studies, particularly MYCN amplification (11).

Due to the multimodal nature of NB treatment, high-risk patients undergo intensive chemotherapy regimens aimed at improving survival. However, current overall survival rates for this group remain below 50% (12).

Treatment for high-risk patients consists of three phases, each with specific objectives. The first phase, induction, aims to achieve maximal reduction of tumor burden and metastasis using five to six cycles of chemotherapy involving various drug combinations. These protocols may follow either North American or European approaches and are followed by surgical resection when feasible (13). The second phase, consolidation and rescue, involves autologous hematopoietic stem cell transplantation (HSCT) to eliminate resistant tumor clones and prevent disease progression. Finally, the post-consolidation phase focuses on treating minimal residual disease and preventing relapse (14). Studies have demonstrated significant improvements in event-free survival and overall survival among high-risk patients who undergo autologous HSCT, particularly tandem transplants. Nevertheless, relapse remains a significant concern despite aggressive treatment strategies (15). As a result, novel targeted therapies have been introduced, offering lower toxicity and reduced morbidity for high-risk patients.

## Targeted immunotherapy vs. neuroblastoma

Neuroblastoma cells express glycopeptide antigens known as gangliosides, which has driven the development of targeted immunotherapies such as monoclonal antibodies (mAbs) against these antigens. The rationale for this approach lies in the fact that gangliosides—particularly those from the b-series—are minimally expressed in mature peripheral autonomic tissue after fetal development (16). Unlikely, neuroblastoma cells demonstrate high expression levels. Notably, GD2 expression has been implicated in tumor adhesion and invasion potential, thus promoting metastasis. Based on this knowledge, targeted therapies have been developed (17).

Several anti-GD2 strategies have been established to attack neuroblastoma cells. Neuroblastomas are often infiltrated by macrophages; anti-GD2 antibodies engage complement component C1q, triggering complement-mediated lysis of neuroblastoma cells (18). Another cytotoxic mechanism involves antibody-dependent cellular cytotoxicity (ADCC), which activates natural killer (NK) cells through Fc receptor engagement (19). However, studies have shown that NK cell activation alone is insufficient for complete destruction of neuroblastoma cells. Therefore, current research focuses on the expression of calreticulin, a molecule that enhances macrophage-mediated phagocytosis when combined with anti-GD2 therapy (20). One advantage of immunotherapy is the widespread expression of glycolipid antigens by neuroblastoma cells. Anti-GD2 antibodies exert their anticancer effects through both complement activation (via C1q interaction) and ADCC mechanisms mediated by NK cell activation.

Currently, various immunotherapeutic agents are used during the post-consolidation phase. Immunotherapy—including anti-GD2 chimeric monoclonal antibodies combined with isotretinoin—has been shown to improve 2-year survival rates up to 66%, compared to 46% with isotretinoin alone (21).

## Evidence related to targeted therapy with naxitamab

A study evaluating the potential sites of residual disease in patients with relapsed or refractory HR-NB included 22 children with a median age of 5 years. Among them, 95% had received prior chemotherapy, 91% had undergone surgery, 36% received radiotherapy, and 18% had previously been treated with anti-GD2 therapy. Unfavorable histology was present in 64% of patients, MYCN amplification in 14%, and 86% were diagnosed at advanced stages (22).

Patients treated with targeted immunotherapy received two or more cycles depending on their response, spaced four weeks apart. Each cycle consisted of granulocyte-macrophage colony-stimulating factor (GM-CSF, 250 μg/m²/day) administered for four consecutive days, along with Naxitamab (3 mg/kg/day) given on three alternate days. All participants were evaluated after the second cycle and subsequently every 2–3 months through imaging and histological studies (23).

The results were encouraging: 68.2% of patients showed a favorable response (partial or complete) to treatment with Naxitamab and GM-CSF. Among the total population evaluated, 59.1% achieved a complete response. Bone disease resolved in 65% of cases, and a similar outcome was observed in 78% of patients with bone marrow involvement.

Naxitamab represents a valuable outpatient treatment option for patients with relapsed or refractory high-risk neuroblastoma. It is an effective and generally safe therapy for this patient population (24). However, it can still be associated with adverse events, although these are generally less severe than those caused by conventional chemotherapy.

**Table 2** provides further information on the side effects related to immunotherapy administration and suggests management strategies before and after infusion (25). Preventive measures may include antihistamines or corticosteroid premedication to reduce the risk of adverse reactions.

**Table 2:** Practical strategies for the efficient management of side effects associated with the use of anti-GD2 naxitamab in patients with high-risk refractory or relapsed neuroblastoma (NB).

Type of side effect	Clinical Manifestactions	Premedication (30 to 60 minutes prior to infusion)	Supportive therapies (as needed)	Caregiver or nurse actions	Frequency of drug administration and timing
**Dermatologic**	Erythema, pruritus, urticaria	Oral cetirizine (2.5 to 10 mg) Oral H2 blocker (2mg/kg)	Intravenous dexchlorpheniramine (0.15mg/kg) Intravenous or oral hydroxyzine (0.5 to 1 mg/kg	Local application of ice for pain of itch relief	IMMEDIATE Every 24 hours
**Cardiovascular**	Hypotension	Intravenous saline bolus	Saline bolus Intravenous naloxone (1μ/kg; repeat every 2 to a 3 minutes) Intramuscular or intravenous epinephrine (0.1 mg/kg)	Patient in Trendelenburg position Intravenous application at peripheral sites requiring more fluids	IMMEDIATE Upon patient assessment, a maximum of two prior to staring vasoactive therapy
	Hypertension	-	Oral enalapril (star at 0.08mg/kg/day)	-	IMMEDIATE Every 12-8 hours
**Respiratory**	Bronchospasm	–	Nebulization with salbutamol: <20 kg (2.5 mg), >20kg (5.0 mg) If necessary Nebulization with ipratropium bromide: <10 kg(125mg), 10-30 kg (250mg), >30 kg (500mg)	Patient in the correct position for effective Nebulization	IMMEDIATE Series of three, with a 20-minute interval Later, schedule every 6-12 hours
	Apnea	-	Intravenous naloxone (1mg/kg; repeat every 2 to 3 minutes)	Patient in the correct position for effective assisted ventilation	IMMEDIATE Repeat every 2 to 3 minutes, assessing the response
	Laryngitis of laryngotracheitis	–	Nebulization with epinephrine (0.5 mg/kg/dose; maximum: 5mg)	Patient in the correct position for effective nebulization	IMMEDIATE Every 3-5 minutes
**Anaphylactic**	Cardiovascular or airway involvement	Oral paracetamol (15 mg/kg) Oral gabapentin (5 to 10 mg/kg every 8 hours. maximum: 600 mg) Intravenous morphine chloride (25 to 100 mg/kg)	Intravenous morphine chloride (25 to 100 mg/kg)	Apply cold or heat, depending on patient tolerance, to painful areas Oxygen mask may be helpful	IMMEDIATE Frequency to assess the patient's hemodynamic status, with an interval of 3-5 minutes between each bolus
**Pain-related**	Standard analgesic measures during naxitamab infusion	–	Intravenous metamizole (30 mg/kg/dose; maximum: 2 g/dose) Intravenous dexketoprofen (1 mg/kg/dose: maximum: 50 mg/dose)	–	BEFORE THE INFUSION Every 6-8 hours
	Residual pain after naxitamab infusion	-	-	-	BEFORE THE INFUSION Every 6-<8 hours
**Gastrointestinal**	Nausea	Intravenous ondansetron (5 mg/m2) Intravenous fosaprepitant on the first day of chemotherapy (5 mg/kg; maximum: 150 mg)	–	–	PRIOR TO ADMINISTRATION Every 6-8 hours
**Others**	Anxiety, nausea	Intravenous lorazepam (0.01 to 0.02 mg/kg) Oral or intravenous lorazepam (0.01 to 0.02 mg/kg. maximum: 1 mg)	-	-	PRIOR TO ADMINISTRATION Every 24 hours

Pain management in opioid-resistant patients					
Administration	Midazolam	Lidocaine	Atropine	Ketamine	
**Before naxitamab infusion**	Bolus (0.05 mg/kg)	≤40 0 >40 kg: bolus	Bolus (0.005 mg/kg)	Bolus (0.5 mg/kg)	
**During naxitamab infusion**		Minute 15: Additional bolus	As needed: Additional bolus Minute 15: bolus (1 a 2 mg/kg) Add up to a total of 4 mg/kg		

The most common side effects include pain (72%), hypotension (60%), urticaria (40%), bronchospasm (28%), decreased level of consciousness (16%), and abdominal pain/discomfort (12%). A small number of patients experienced grade IV adverse events, such as anaphylactic reactions (4 episodes), fever, and respiratory depression (one case each); however, these events did not require discontinuation of treatment.

The implementation of targeted therapy in neuroblastoma offers multiple benefits for patients with relapse or refractory disease, not only by improving overall and event-free survival, but also by reducing treatment-related toxicity. Given that the majority of neuroblastoma patients in Mexico are classified as high-risk, the incorporation of these novel therapies should be a priority in national treatment protocols to ensure better survival outcomes.

## Case report and informed consent for naxitamab therapy

It is important to highlight that, prior to initiating Naxitamab therapy, both of the patient’s parents signed an informed consent document. They were thoroughly informed about the drug’s availability, potential adverse effects, and expected benefits. They were also made aware that their child would be the first patient in Mexico to receive this therapy.

## Case presentation

We present herein the case of a two-year-old male patient from Mexico City, without any relevant past medical history. He was admitted due to a three-month history of left fronto-temporal mass and left exophthalmos. On examination, the patient showed no signs of fever, asthenia, weakness, or weight loss.

Physical examination revealed an exostosis in the left fronto-parietal region of the skull, which was non-tender and hard in consistency. The face appeared asymmetric, with exophthalmos of the left eye, associated with left-sided proptosis, with upper eyelid ptosis and erythema. No other abnormalities were found during the examination.

## Diagnostic imaging studies

**Figure 1:**
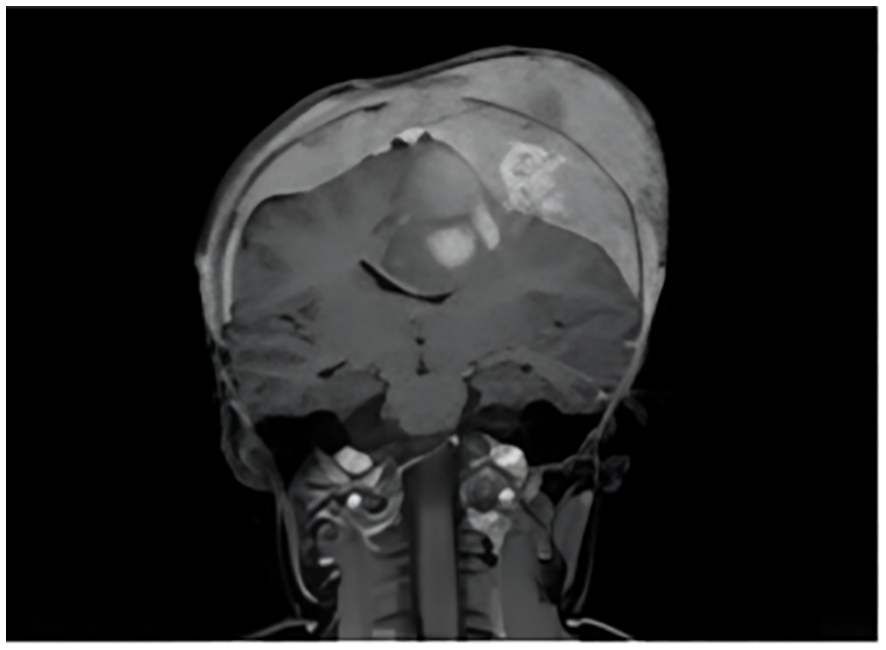
Contrast-enhanced whole-body MRI showed an expansive and infiltrative lesion in the left parietal region.

A whole-body contrast-enhanced MRI revealed a lobulated, ill-defined mass at the right retroperitoneal and paravertebral region, located between the aorta and the liver, partially adjacent to the inferior vena cava, without evidence of infiltration. The mass encased and slightly displaced the contours of the superior mesenteric vein and the celiac trunk, without apparent involvement. Additionally, there was a lesion (**Figure 1**) with an expansive and infiltrative appearance, showing loss of both outer and inner cortical bone layers of the cranial vault, with tendency to form a sunburst-type periosteal reaction. The loss of cortical definition was associated with increased soft tissue volume in the parietal region, partially extending into the temporal region and lateral aspect of the left orbit, causing proptosis and a mass effect on the brain parenchyma. These findings were consistent with metastatic neuroblastoma (T4).

A PET-CT with octreotide revealed focal uptake of the somatostatin analog as a multi-lobulated retroperitoneal lesion, with coarse calcifications, arising from the right adrenal gland, measuring 49.8 x 40.3 x 56 mm, with a maximum standardized uptake value (SUVmax) of 6.2, suggestive of a primary tumor (neuroblastoma). Additionally, there was an expansive lesion at the left greater wing of the sphenoid bone, measuring 47 x 74 mm, with an SUVmax of 5.9, displacing the ocular globe. Another lesion was identified at the left lateral ventricle, located toward the roof and posterior horn, measuring 21 x 35 mm, with an SUVmax of 2.0. There were also findings of widespread dissemination to the bone marrow, compatible with secondary deposits.

**Figure 2:**
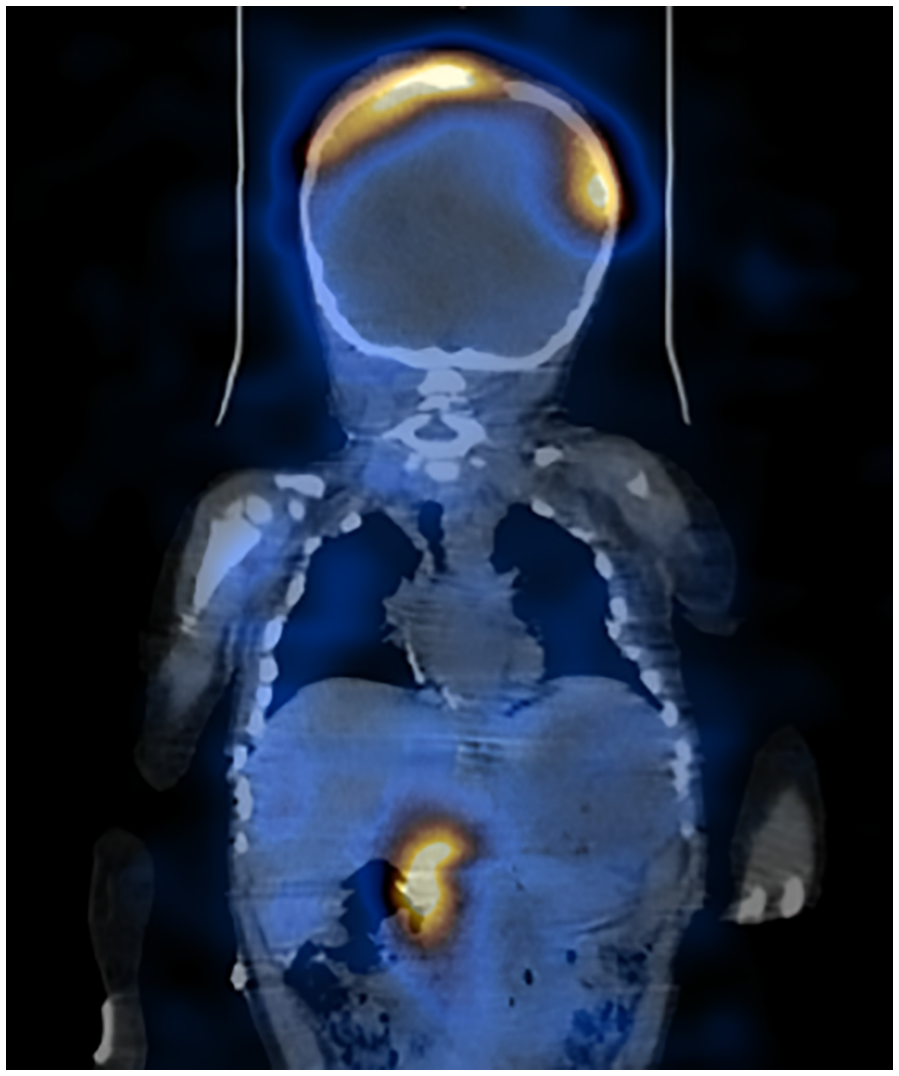
Meta-iodobenzylguanidine shows a primary lesion in the right adrenal gland and metastatic sites in the cranial vault and intra-axial lesions.

As part of the diagnostic work-up, an iodine-123 metaiodobenzylguanidine (MIBG) scan (**Figure 2**) was performed, showing findings consistent with neural crest-derived neoplastic tissue in the right adrenal gland, measuring 49.8 x 40.3 x 56 mm. Lytic lesions with soft tissue involvement were identified in the skull, creating a mass effect of 10.8 x 43.5 mm involving the entire cranial vault, with extension into the sphenoid bone, infiltrating the left orbit and displacing the ocular globe anteriorly. Additionally, a supratentorial, intra-axial lesion was observed within the left lateral ventricle, extending to its roof and posterior horn, measuring 21 x 35 mm on the axial plane, with increased intraventricular volume and right-sided midline displacement, along with generalized bone marrow infiltration.

## Patient staging

The patient was classified as high risk, meeting the criteria of metastatic disease and being older than 18 months of age. Due to this high-risk status, a three-phase therapy plan was determined: induction, consolidation with stem cell transplant, and maintenance therapy using specific agents such as anti-GD2 monoclonal antibodies. However, in this patient, due to poor response to conventional chemotherapy, it was decided to initiate anti-GD2 therapy prior to transplant consolidation.

## Treatment

High-risk induction therapy was initiated, consisting of five chemotherapy cycles prior to resection of the primary tumor. Two chemotherapy regimens were alternated. The first included two cycles of carboplatin 450 mg/m² on day 1, ifosfamide 1800 mg/m² on days 1–5, and etoposide 100 mg/m² on days 1–5. The second included two cycles of cisplatin 90 mg/m² on day 1, cyclophosphamide 500 mg/m² on days 1 and 2, and doxorubicin 45 mg/m² on day 1. A fifth cycle consisted of carboplatin 450 mg/m² on day 1, ifosfamide 1800 mg/m² on days 1 and 5, and etoposide 100 mg/m² on days 1 and 5.

A partial resection of approximately 85% of the primary tumor was then performed, followed by two additional alternating cycles of systemic chemotherapy prior to radiotherapy. Radiation therapy was administered to the cranial area and the residual retroperitoneal tumor at a dose of 21.56 Gy. A follow-up PET-CT with octreotide showed a partial response in both bone and soft tissue metastatic lesions. Thus, the patient was considered a candidate for anti-GD2 immunotherapy with naxitamab.

Based on imaging studies, the patient was deemed to have shown a poor response to conventional chemotherapy. As the neuroblastoma was considered refractory, immunotherapy with naxitamab was initiated.

The patient was enrolled in the “HITS-17-251” protocol, receiving naxitamab in combination with systemic chemotherapy consisting of irinotecan and temozolomide. Within the HITS protocol, naxitamab was combined with conventional chemotherapy due to inadequate response in both soft tissue and bone metastatic disease.

The administered dose of naxitamab per cycle was 9 mg/kg, divided into four doses of 2.25 mg/kg on days 2, 4, 8, and 10. Temozolomide was given at 150 mg/m² on days 1 to 5, and irinotecan at 50 mg/m² on days 1 to 5, with cycles repeated every 21 days.

## Adverse effects of immunotherapy

A total of five cycles were administered. The adverse effects reported in this patient following immunotherapy were grade I–II and mild during the first four courses. This included erythema, pruritus, and urticaria, all of which were managed with systemic antihistamines and resolved uneventfully. The patient also experienced hypotension, which resolved with intravenous fluid administration.

After the fifth and final cycle, the patient exhibited a favorable clinical response of the cranial metastatic disease, with complete response confirmed on imaging following the last cycle of naxitamab (**Table 3**).

**Table 3:** Evolution of tumor size at diagnosis and after therapy with naxitamab.

STUDY	TUMOR SIZE
**PET-CT for diagnosis for diagnosis**	➔**ADRENAL GLAND TUMOR RIGHT** 49.8 X 40.3 X 56 MM SUB MAX 9.0 ➔**SUPRATENTORIAL INTRA-AXIAL LESION LOCATED WITHIN THE LEFT LATERAL VENTRICLE** 21X35 MM SUB MAX 5.9
**PET-CT for After Immunotherapy**	➔**ADRENAL GLAND TUMOR RIGHT** 28.4 x 29.2 SUB MAX 3.5 ➔**SUPRATENTORIAL INTRA-AXIAL LESION LOCATED WITHIN THE LEFT LATERAL VENTRICLE** 3.5X5 MM SUB MAX 2.0 REDUCTION IN THE SIZE OF THE PRIMARY TUMOR BY 50% AND IN THE SITE OF METASTASIS BY MORE THAN 80%, WITH A REDUCTION IN THE METABOLISM OF THE LESIONS

The anatomical areas of the primary tumor and the metastatic area that had the greatest changes in size after the use of immunotherapy are highlighted in bold.

Given this positive response, consolidation with tandem autologous hematopoietic stem cell transplantation was planned. However, After the fifth cycle of Naxitamab, the patient developed progressive neurological deterioration, characterized by increasing headaches, generalized tonic-clonic seizures, and worsening neurological status. As a result, he required ventilatory support. Brain MRI revealed demyelination in the basal ganglia and cerebellum, and hydrocephalus. Infectious processes and tumor progression were discarded. Magnetic resonance angiography showed findings consistent with vasculitis. Consequently, the patient’s neurological deterioration was attributed to immune-related neurotoxicity secondary to immunotherapy.

The patient required advanced airway management and intensive care support for 27 days in the pediatric intensive care unit. Two cycles of high-dose methyl prednisolone, 10 mg/kg dose, and hyperimmune gamma globulin were administered to counteract inflammation and toxicity. Unfortunately, clinical progression was unfavorable, and the patient developed brain death secondary to severe cerebral edema.

## Discussion

Immunotherapy is considered an innovative treatment strategy for pediatric cancer. Immunotherapeutic approaches can be classified by mechanism of action and include cytokines, monoclonal antibodies, cellular immunotherapy, chimeric antigen receptor (CAR) T-cell therapy, immune checkpoint inhibitors, and genetically modified oncolytic viruses (26).

In neuroblastoma, the most studied form of immunotherapy involves monoclonal antibodies. The Children’s Oncology Group (COG ANBL0032) conducted a randomized phase III trial demonstrating improved survival in high-risk neuroblastoma patients treated with the anti-GD2 antibody ch14.18 (dinutuximab). Patients with metastatic disease and MYCN amplification who received standard chemotherapy, surgery, local radiotherapy, and autologous stem cell transplantation were randomized to receive isotretinoin alone or in combination with dinutuximab. The addition of dinutuximab significantly improved 2-year event-free survival (EFS) (66% vs. 46%, p = 0.01) and overall survival (OS) (86% vs. 75%, p = 0.02) (27).

These findings support the important role of immunotherapy in patients with poor response to conventional chemotherapy, improving both EFS and OS. High-risk patients often have poor outcomes due to primary refractory disease or relapse.

Galassi et al. explored mitochondrial damage and activation of stress pathways that sensitize neuroblastoma cells to naxitamab. Currently, international protocols already incorporate immunotherapy as first-line treatment for high-risk patients, during the post-consolidation phase following autologous stem cell transplantation (28).

Naxitamab is a humanized monoclonal antibody (hu3f8) targeting GD2, used in the present case as part of the HITS protocol due to partial response to chemotherapy. Modern oncologic strategies aim for precision medicine and targeted therapies not only to increase survival and reduce relapse but also, to minimize long-term treatment-related sequelae. In a study by Mora et al., 73 high-risk neuroblastoma patients in first or second complete remission received 385 total cycles of naxitamab. The reported 3-year EFS was 58% and 3-year OS was 82%, representing a promising alternative to improve outcomes in this patient population, who have failed to achieve remission with multiple conventional therapies. In comparison, the patient presented here also responded well to immunotherapy, particularly in contrast to the limited efficacy of prior chemotherapy (29).

An additional benefit of immunotherapy is the reduced toxicity compared to conventional chemotherapy. According to previous European experience with naxitamab, with adequate premedication and timely identification, adverse events were properly managed on an outpatient basis. Common adverse events included hypotension (98%), pain (96%), rash (83%), fever (79%), vomiting (52%), and nausea (50%) (30). These events were low-grade and none contributed to increased mortality when appropriately managed.

In the present case, the patient experienced low-grade adverse events during the first four cycles, including hypotension managed with fluids (no vasopressors required), and pain controlled with buprenorphine infusions, which decreased in intensity during subsequent cycles. In the last two cycles, he developed a rash that resolved with antihistamines without progressing to anaphylaxis.

However, after the fifth cycle, although the patient initially developed only a mild rash, one month later he presented signs of neurotoxicity, which ultimately led to fatal complications.

While immunotherapy is not novel in high-income countries, its use in Latin America remains limited due to high associated costs. This patient was the first to receive anti-GD2 immunotherapy at the National Institute of Pediatrics in Mexico, as part of an initial response strategy, rather than as second-line treatment after relapse.

Despite the low incidence of neuroblastoma in Mexico, a high percentage of cases are diagnosed as high risk. As a result, survival rates remain below 45% for this group. Expanding access to specific immunotherapies such as naxitamab in Mexican health centers could improve outcomes. However, two key challenges must be addressed: first, the development of regional consensus to ensure molecular diagnostics are available to all patients; and second, the establishment of clinical trials to standardize and evaluate immunotherapy outcomes, generating national evidence to support widespread implementation.

Although this is a unique case, and its unfavorable outcome may discourage the use of such therapy, the excellent disease response prior to the development of neurotoxicity underscores the potential value of naxitamab. Therefore, broader use of such therapies in Mexico should be accompanied by robust clinical evidence and systematic planning.

## Conclusions

Immunotherapy represents an innovative strategy in the treatment of pediatric cancers, including HR-NB. The COG ANBL0032 phase III trial demonstrated significant improvement in event-free and overall survival with anti-GD2 antibody (dinutuximab) following consolidation in patients with HR-NB. Naxitamab, a humanized anti-GD2 antibody, offers outpatient administration and demonstrated favorable response rates in patients with relapsed/refractory NB. In this case, naxitamab induced a complete radiological response after partial response to chemotherapy, supporting its role in chemo-resistant disease. Adverse effects were mostly mild and manageable, although in this particular case, fatal neurotoxicity occurred following therapy. While this case highlights the potential of immunotherapy, it also underscores challenges in toxicity monitoring and access. Cost remains a major barrier. Implementation in Mexico is further hindered by limited molecular diagnostics and lack of standardized protocols. Nevertheless, this case marks a milestone for naxitamab use in Mexican pediatric oncology patients.

In addition to improving event-free (EFS) and overall survival (OS) in patients diagnosed with high-risk neuroblastoma, the primary goal of implementing novel immunotherapeutic strategies is to reduce adverse events and decrease both, short and long-term mortality.

It is worth noting that the current cost of anti-GD2 therapies available in Latin America is estimated to exceed $450,000 USD. In a country like Mexico, where the minimum daily wage is approximately $10 USD (31), access to these therapies remains unattainable for the majority of patients. Therefore, it is essential to highlight the importance and substantial clinical impact of immunotherapy on survival outcomes in patients with refractory neuroblastoma. This recognition should support advocacy for broader access to these therapies. Despite their high costs, the demonstrated benefits should outweigh the disadvantages, justifying efforts to make them accessible to all patient populations.

Future studies in Mexico and Latin America must focus on optimizing dosing strategies, minimize adverse effects, and improve survival outcomes and quality of life for patients receiving immunotherapy.
